# Epigenomics of Total Acute Sleep Deprivation in Relation to Genome-Wide DNA Methylation Profiles and RNA Expression

**DOI:** 10.1089/omi.2016.0041

**Published:** 2016-06-01

**Authors:** Emil K. Nilsson, Adrian E. Boström, Jessica Mwinyi, Helgi B. Schiöth

**Affiliations:** Department of Neuroscience, Uppsala University, Uppsala, Sweden.

## Abstract

Despite an established link between sleep deprivation and epigenetic processes in humans, it remains unclear to what extent sleep deprivation modulates DNA methylation. We performed a within-subject randomized blinded study with 16 healthy subjects to examine the effect of one night of total sleep deprivation (TSD) on the genome-wide methylation profile in blood compared with that in normal sleep. Genome-wide differences in methylation between both conditions were assessed by applying a paired regression model that corrected for monocyte subpopulations. In addition, the correlations between the methylation of genes detected to be modulated by TSD and gene expression were examined in a separate, publicly available cohort of 10 healthy male donors (E-GEOD-49065). Sleep deprivation significantly affected the DNA methylation profile both independently and in dependency of shifts in monocyte composition. Our study detected differential methylation of 269 probes. Notably, one CpG site was located 69 bp upstream of *ING5*, which has been shown to be differentially expressed after sleep deprivation. Gene set enrichment analysis detected the Notch and Wnt signaling pathways to be enriched among the differentially methylated genes. These results provide evidence that total acute sleep deprivation alters the methylation profile in healthy human subjects. This is, to our knowledge, the first study that systematically investigated the impact of total acute sleep deprivation on genome-wide DNA methylation profiles in blood and related the epigenomic findings to the expression data.

## Introduction

The architecture of sleep in humans is divided into two major types, nonrapid eye movement and rapid eye movement sleep. These two types of sleep have been associated with specific brain functions as well as physiological functions, such as eye movements or muscle tone. Furthermore, sleep has a strong impact on the endocrine system, influencing the levels of, for example, the thyroid hormone, growth hormones, and melatonin. It is, therefore, not surprising that sleep disturbances have been linked to a multitude of disorders, including both cognitive and metabolic impairments (Qureshi and Mehler, 2014).

Partial or total sleep deprivation (TSD) has been shown to affect cellular and metabolic functions such as neutrophil maturation (Christoffersson et al., 2014), the production of cytokines (Irwin et al., 2006), and neuron-specific enolase and calcium binding protein B levels, which can be indicative of an impaired functionality of the blood–brain barrier or neuronal damage (Benedict et al., 2014). Recently, a genome-wide transcriptomics study has revealed that 75% of the transcripts tested had their transcription phase aligned with the plasma levels of melatonin, a reliable marker of circadian rhythmicity (Möller-Levet et al., 2013). The authors of that study have compared the expression profiles of subjects under sleep restriction (6-h sleep opportunity per night) and expression profiles of subjects under normal sleep conditions (10-h sleep opportunity per night).

DNA methylation patterns can be inherited or changed depending on environmental influences on the human organism. At the same time, it is been shown that DNA methylation patterns within the first 2000 bp upstream or downstream of the transcription start site (TSS) have the highest impact on gene expression (Wagner et al., 2014). It can, therefore, be hypothesized that DNA methylation of CpG dinucleotides may play a significant role in the regulation of the observed expressional changes.

Some of the genes governing the sleep–wake cycle, for example, the genes connected to *CLOCK/BMAL1*, are, in part, regulated by DNA methylation (Doi et al., 2006). The importance of DNA methylation in this context is highlighted by the fact that sleep deprivation increases the expression of DNA methyltransferases and members of the methionine transferase complex (Massart et al., 2014) in mice.

Although Cedernaes et al. (2015) have recently studied the effect of sleep deprivation on DNA methylation of core clock genes (BMAL1, CLOCK, CRY1, PER1), no study has yet addressed the question of to what extent do genome-wide methylation changes occur as a result of disrupting the sleep–wake cycle in humans. In this study, we aimed to investigate the impact of 24 h TSD on the genome-wide methylation profile of whole blood in human subjects using a randomized, single-blinded, and crossover trial design. The participants, not the observers, were blinded until the evening of the intervention and the participants were also unaware that they were in a crossover trial.

## Materials and Methods

### Subjects

Sixteen healthy white Caucasian male volunteers (age: 23.3 ± 3.4) participated in the study. All subjects reported normal sleeping behavior and sleep–wake rhythm before the experiment. Reports of smoking, use of medication, or a history of endocrine or psychiatric disorders resulted in an exclusion from the study. Study participants underwent both a physical examination and a routine laboratory value examination by the Department of Clinical Chemistry and Pharmacology (Uppsala, Sweden). This included measurements of C-reactive protein concentrations (normal value <6 mg/mL) and white cell blood counts (normal value 5.2 ± 0.7) to exclude any ongoing acute illness before study start. Written informed consent was obtained from all participants and the study was approved by the regional ethical committee in Uppsala, Sweden. The study has been registered at ClinicalTrials.gov (accession number NCT01730742).

### Study design and procedure

The samples used in this study have been collected in the frame of the previously completed works (Benedict et al., 2014; Christoffersson et al., 2014). Each participant completed two study sessions, which included a phase of total acute sleep deprivation and a phase of normal sleep, in a randomized blinded crossover design.

**FIG. 1.**
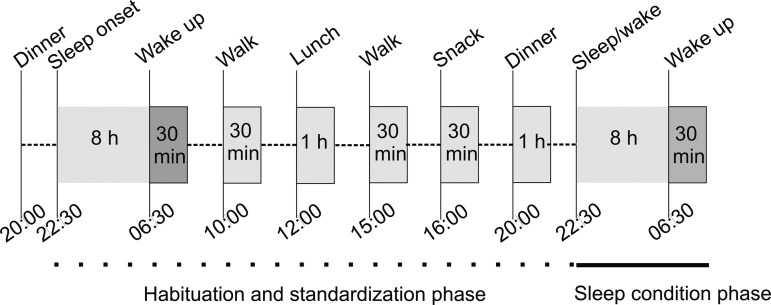
Study design overview: Schematic overview of the study design investigating TSD-induced changes in the epigenome. Subjects participated in two study sessions comprising two nights, that is, the habituation night and the condition night. Meals and the amount of exercise were controlled to assure energy homeostasis for each individual. Depending on the study arm, patients underwent either TSD or had a normal period of sleep (control arm) during the second night. The sampling times were 06:30 on both mornings as indicated by a *darker shade of gray* in the picture. TSD, total sleep deprivation.

Each session was composed of an initial 28.5 h long baseline period under which the included subjects were eating, sleeping, and performing light exercises under standardized conditions (Fig. 1). The exercises consisted of two 30-min walks during the day and were implemented to simulate normal conditions. Water was provided *ad libitum* throughout the study. Subjects arrived fasting in the evening at 20:00 in the study center to the habituation phase and spent one night sleeping under laboratory conditions. Participants were single blinded to the condition (i.e., they did not know whether they will have the possibility to sleep for 8 h or not) until 4 h before the sleep intervention. Blood glucose, CRP, and monocyte subpopulations were monitored every 12 h throughout the session, at 06:30 and 18:30. This was primarily done to ensure compliance to the study restrictions on food and elevated inflammatory status. The samples used for microarray analysis were those taken at 06:30 on the morning after the intervention.

The determination of blood parameters was performed by the Department of Clinical Chemistry and Pharmacology at Uppsala University Hospital. Blood cell counts were determined by standard histology, where the different cell types are stained and smeared on a slide before they are counted. Sleep quality and length were assessed by polysomnography (Embla A10; Flaga, hf, Reykjavik, Iceland) that consisted of electroencephalography, electrooculography, and electromyography. Wakefulness was monitored by a handler. On the morning after the intervention, peripheral blood was collected in EDTA tubes and immediately frozen by means of a 50:50 mixture of ethanol and dry ice.

### DNA extraction and methylation profiling

Genomic DNA was extracted using robot-assisted phenol/chloroform extraction at the Latvian Biomedical Research and Study Centre in Riga (BMC), Latvia. The Latvian BMC provides core facilities for molecular and cell biology research for Latvian universities as well as international collaborators. Bisulfite conversion of DNA and hybridization to the Illumina 450K methylation BeadChip (Illumina, San Diego, CA, USA) were performed at the Science for Life Laboratory (SciLifeLab, Uppsala, Sweden), a scientific center run in collaboration with four Swedish universities, that is, the Karolinska Institutet, KTH Royal Institute of Technology, Stockholm University, and Uppsala University. Bisulfite conversion converts unmethylated cytosines to uracil at a high conversion rate (>99%). However, it does not differentiate between CpG methylation and other DNA-based epigenetic marks such as non-CpG methylation or 5′ hydroxymethylation.

Before hybridization to the chip, DNA was whole genome amplified, enzymatically fragmented, precipitated, and resuspended. After hybridization to the array overnight at 48°C, C to T base exchanges were detected by single-base primer extension. The 16 pairs of samples were distributed across three 12-sample physical chips. We used duplicate samples for two subjects, resulting in four pairs of technical replicates. The samples are placed on the chips in pairs so that samples from the same individual are next to each other. This strategy is based on the results by Buhule et al. Figure 1, sample 2. The two technical replicate pairs were placed on chips 2 and 3 (Buhule et al., 2014). This strategy minimizes chip batch effects and allows for more robust analysis.

### Reading and preprocessing of Illumina 450K data

The R package (Team, 2012) methylumi (Gentleman et al., 2004) was used to read the intensity data files and to produce beta values. Beta values range from 0 to 1 depending on the degree of methylation at a specific locus. Since the density distribution of these values suffers from severe heteroscedasticity, thereby making them unsuitable for downstream analysis, the beta values were transformed to M values, which are statistically more robust (Du et al., 2010).

All sample mean normalization (R function “asmn”) was chosen instead of the first sample normalization used in the software package GenomeStudio^®^ (Yousefi et al., 2013) because of improvements in replicate comparability. This step was performed to equalize the overall intensity across all subjects. In addition, the original annotation supplied by Illumina was used together with extended annotation (Price et al., 2013) to exclude probes meeting any of the following criteria: at least one single nucleotide polymorphism (SNP) in probe region, matched multiple genomic regions, or targeted sex chromosomes. Probes with a detection *p* value >10^−5^ were excluded as well. This filtering step resulted in 317 366 eligible probes (65% of probes on the array) to be used in downstream analyses.

To further ensure the detection of methylation shifts with a relevant impact on expression levels, the analysis was restricted to probes located within a region 2000 bps upstream and downstream from the TSS. This cut-off value was introduced by Wagner et al., who showed that variation in methylation and expression is most closely related to this area (Wagner et al., 2014). The final number of probes used in the analysis amounted to 167,490.

Probes were subsequently subjected to quantile normalization followed by beta mixture quantile normalization (Teschendorff et al., 2013). This normalization step was then applied to minimize the bias caused by the two different types of probes (type 1 and type 2) and to reduce batch effects. Hierarchical clustering and multidimensional scaling were used to determine whether there were any outliers, which resulted in the exclusion of one subject and, thus, emerging at a final number of 30 samples in 15 pairs.

### Statistical analysis

The statistical analysis comprises two steps. Initially, a paired analysis was performed on all probes to identify differentially methylated CpG sites depending on sleep deprivation. In this analysis, the ratio of neutrophils to leukocytes was used as a covariate (see [Eq. 1]). Applying this equation allows us to estimate the influence of TSD and neutrophil/leukocyte (N/L) ratio separately by taking their respective coefficients into consideration.

\begin{align*}Mvalue = Intercept + a*Condition + b* { \frac {
\left[ { Neutrophil } \right] }  { \left[ { Leukocytes } \right] }
} \qquad \qquad \qquad \qquad {[\rm Eq. 1]}\end{align*}

Equation 1: The statistical model used in step I of the analysis. The model is applied in a paired design where t-statistics and magnitude of change are calculated based on 15 pairs of differences.

**FIG. 2.**
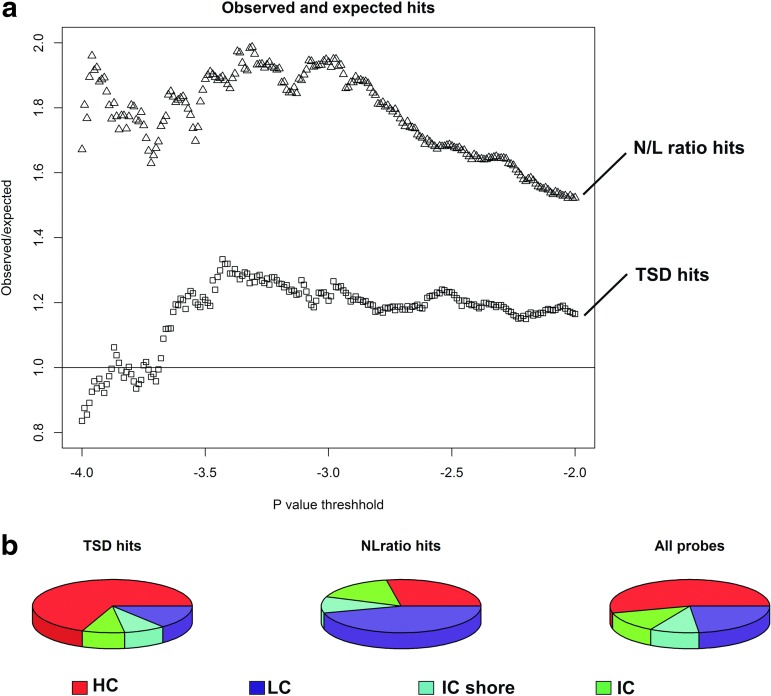
Observed and expected number of hits for TSD and the N/L ratio as well as their CpG density distribution: Ratios between observed and expected changes in methylation with dependence on TSD at different *p* value thresholds **(a)**. The upper line is determined by the number of significant b coefficients from [Eq. 1] and the lower line depends on the number of significant a coefficients. The log (*p* value) threshold for entry in analysis step II (testing whether an observed methylation change is larger than the median change observed for technical replicates) was defined as 2.5. A total of 652 probes for TSD (coefficient a) and 891 for the N/L ratio (coefficient b) passed this threshold. Distribution of the probes among different CpG density classes **(b)**. HC probes are defined as having a CG base content more than 55%, Obs/Exp CpG ratio more than 0.75, and being longer than 500 bps. The corresponding criteria for IC probes are 50%, 0.48, and 200, respectively, whereas those probes that did not fit either criteria were labeled as LC. ICshore probes are those that are located at the end of an IC-Island (Price, et al., 2013). The left circle diagram represents the distribution of the 652 probes associated with sleep deprivation and the middle diagram shows the N/L ratio-associated probes. The right diagram displays the background distribution of all investigated probes. Chi-squared distribution tests determined that TSD-related probes (probes with significant a coefficients) were more likely to be HC probes. N/L ratio-related probes (probes with significant b coefficients) were more likely to be LC probes. HC, high-density CpG; IC, intermediate-density CpG; ICshore, shore region of intermediate-density islands; LC, low-density CpG; N/L, neutrophil/leukocyte.

The R library Limma (Smyth, 2005) was used for the application of the model followed by parameter adjustment with the treat command. To account for technical variability, the Limma-specific function, duplicate correlation, was used with the four technical replicate pairs as input variables. The ratio of observed and expected probes for different *p* value thresholds was used to determine the optimal *p* value threshold for our data (step I, see Fig. 2a). The number of expected false positives was calculated as the number of probes being tested multiplied by the *p* value threshold. In step II, a *t*-test was performed for all probes that passed analysis step I to determine whether the observed change in methylation was higher than the median change in technical replicates. In this step, correction for multiple testing was done using the Benjamini–Hochberg method. Finally, proximal probes annotated to the same gene were subjected to uncorrected pair-wise *t*-tests to determine whether multiple probes within the same gene were significantly differentially methylated. For this test, a *p* value <0.05 was considered significant.

### Targeted analysis and methylation–expression correlation

Genes reported to be dependent on time awake by Möller-Levet et al. (2013) were chosen for a targeted analysis. Probes closest to the TSS of genes, matching 1 of the 122 genes reported by Möller-Levet et al., were investigated in the same manner as described for the genome-wide analysis. We also investigated the correlation between methylation and expression by Pearson's correlation analysis in a separate cohort of methylation and expression data in peripheral blood mononuclear cells (PBMCs) from 10 healthy Caucasian male blood donors, aged 30–66 years (E-GEOD-49065) (Steegenga et al., 2014).

### Enrichment, physical interaction networks, and comethylation analysis

Enrichment analyses were performed using ConsensusPath (Kamburov et al., 2011). This analysis included those genes that were associated with one or more probes showing significant changes in methylation depending on TSD. The ConsensusPath performs a hyper geometric test for the detection of overrepresented pathways in sources such as KEGG, OMIM, GeneOntology, or Wikipathways. The analysis was performed using default settings. A *q* value <0.05 was considered significant. A similar hyper geometric test was performed in R for enrichment of different CpG density probes among probes linked to TSD and the N/L ratio. The CpG density was divided into four classes according to Price et al. (2013): high-density CpG (HC), intermediate-density CpG (IC), low-density CpG (LC), and shore region of intermediate-density islands (ICshore). The physical interaction network between genes close to significant probes was examined using GeneMANIA (Mostafavi et al., 2008). Settings were chosen in such a way that they allowed the detection of physical interactions and excluded intermediate nodes. Analysis of comethylation networks was performed on the probes judged most likely to be associated with differential expression in response to TSD.

## Results

### Genome-wide analysis

Figure 2a depicts the ratios between observed and expected number of significant probes correlated with TSD and N/L ratio as obtained in analysis step I. On the basis of these observations, probes with *p* values <10^−2.5^ (corresponding to a ratio maximum of 1.22) were carried forward to subsequent analyses (step II). This corresponds to a peak in the ratio between observed and expected number of probes (Fig. 2a) and maximizes the number of calculated true positives that were carried forward. We discovered 269 probes that were differentially methylated in sleep versus sleep deprivation while taking the median change in technical replicates into account (Supplementary Table S1). Many of these CpG sites were group-wise associated with the same gene, and 119 genes (44%) displayed more than one significantly changed probe (Supplementary Table S1). The raw data used in this publication have been uploaded to ArrayExpress and have the accession number E-MTAB-4664

### Targeted analysis and methylation–expression correlation

One of the 269 CpG sites tested to be differentially methylated in sleep deprivation is located in a HC region 69 bp upstream of the gene *ING5*. In line with our findings, the expression of this gene correlated with time awake, as in the study by Möller-Levet et al., (2013). Apart from *ING5*, the authors also reported 121 other transcripts to be affected by time awake. This corresponded to 640 CpG sites in our data set. Four of these CpG sites showed a significant sleep-dependent difference in methylation in our study (*ING5, USP46, CIT,* and *FGFR1OP2*, Fig. 4). Methylation–expression correlation analyses were also performed in a separate cohort (E-GEOD-49065), consisting of 10 healthy Caucasian male blood donors, aged 30, 31, 34, 35, 43, 52, 62, 64, 65, and 66 years. In addition, for the 237 CpG sites listed in Supplementary Table S1 (88%), both methylation and expression data were available. We detected significant correlations between methylation and expression for 26 CpG sites (labeled with an “*” in Supplementary Table S1).

### Enrichment, physical interaction networks, and comethylation analysis

Enrichment analysis revealed two pathways to be affected by methylation changes, that is, the Notch (represented by the genes *NOTCH4, RING1, HES1, CCND1,* and *MAML1, q* value <0.05) and the Wnt (*CCND1, FZD8, FRAT1, FZD6,* and *WNT4, q* value <0.05, source: Wikipathways (Pico et al., 2008)) signaling pathway. Interestingly, two of these genes displayed significant methylation–expression correlations (*FZD6* and *FRAT1*). The 269 CpG sites differentially methylated with dependence on TSD were mainly located in HC regions (Fig. 2b). Interestingly, the opposite was observed for the N/L ratio correlated probes. Both TSD and the N/L ratio-linked probes displayed a different distribution of CpG island density as compared with the background probes (*p* value <0.05, chi-squared test, Fig. 2b).

Using GeneMANIA, we scrutinized to which extent physical interactions might occur between proteins encoded by genes that are affected by methylation changes. The interaction analysis highlighted the heat shock 7 kDa protein 4 (coding gene *HSPA4*), which physically interacts with eight other proteins encoded by genes listed in Supplementary Table S1 *(CUL5, ZAP70, HES1, DNAJC3, RCOR1, YWHAZ, CSNK2A1,* and *MED6)*. Two of these genes (*DNAJC3* and *MED6*) were significantly correlated with expression (Supplementary Table S1). Although Wagner et.al. (2014) showed that relevant correlations between methylation and expression occur, especially in regions adjacent to the TSS, other authors assume that the relevant connections between methylation and expression observed for CpG sites are located within promoter regions of genes (Fouse et al., 2008). Based on the latter assumption and on our observation that probes related to TSD were primarily located in HC islands, the 100 HC sites within the first 2000 bp upstream of TSS were selected for a methylation–methylation correlation analysis to further investigate comethylation patterns likely to be associated with differential expression in TSD.

**FIG. 3.**
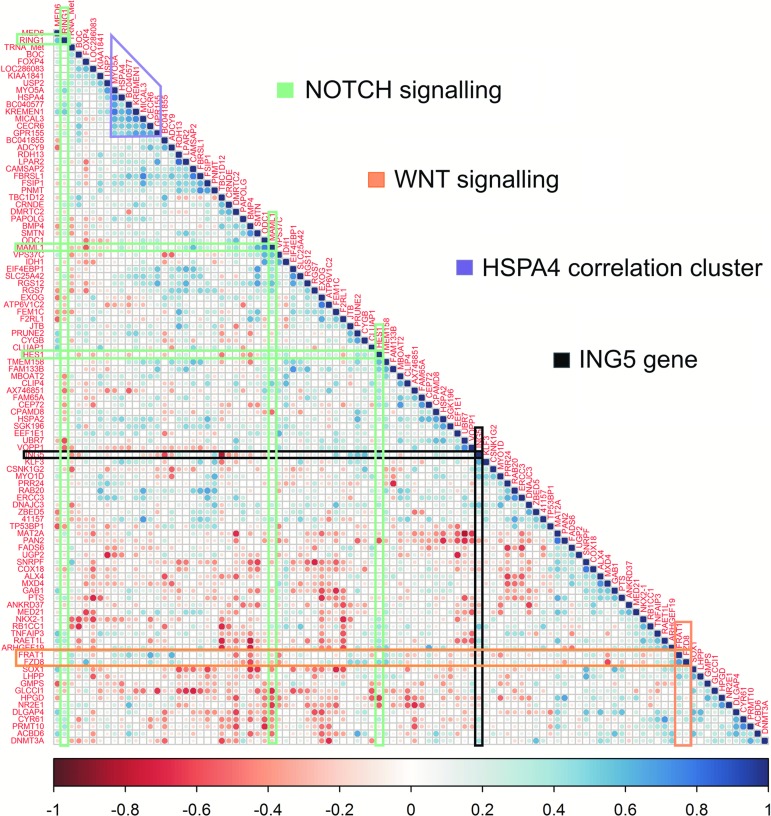
Methylation–methylation correlation of promoter probes: A correlation plot of methylation values in 100 promoter probes associated with TSD. Correlation coefficients were calculated between each pair of probes and were then ordered by hierarchical clustering. This way, the probes with the closest correlation profiles are next to each other in the figure. The correlations range from −1 (negative correlation) to 1 (positive correlation). The genes corresponding to the enriched pathways Notch (*green*) and Wnt (*orange*) are highlighted together with the gene *ING5* (*black*) and the HSPA4 correlation cluster (*purple*).

As shown in Figure 3, three of these CpG sites were located in the promoters of Notch-associated genes *(RING1, MAML1,* and *HES1).* Interestingly, the methylation of CpG sites located in the promoter of Notch genes correlated, to a relevant extent, inversely with the methylation of downstream-located CpG sites *(NOTCH4* and *CCND1)* (mean R = −0.38, SD = 0.06 across all comparisons). Although not significant, the two Wnt pathway-associated promoter CpG sites (located next to each other in the correlation plot, Fig. 3) tended to correlate with probes within promoters of *CCND1, WNT4*, and *FZD6* (*p* values <0.1 for all comparisons).

## Discussion

We show in the present report that total acute sleep deprivation induces significant changes in the genome-wide methylation profile in blood, which is partly independent of the N/L ratio. This is, to our knowledge, the first study that systematically investigated the impact of TSD on the genome-wide DNA methylation profile in blood and related the epigenetic findings to expression data. We detected 269 genes and 184 associated CpG sites that changed in methylation because of TSD.

One of the detected CpG sites found in a differentially methylated state belongs to the gene *ING5*, which has been previously shown to be differentially expressed as a result of longer time awake. *ING5* is a tumor repressor gene and the encoded protein together with TIP60 is able to acetylate p53 at K120 in the DNA binding domain (Liu et al., 2013). *ING5* displays a pattern of decreased methylation in our study and was shown to decrease expression the longer the subject stayed awake (Möller-Levet et al., 2013), pointing to the possibility of a positive correlation between methylation and expression. Our calculations in the independent cohort confirmed this hypothesis with detection of a positive correlation coefficient of 0.28. Disruption of *ING5* function has also been linked to several types of cancers, particularly gastric cancers (Xing et al., 2011; Zheng et al., 2011). Thus, our results lend further support to the observation that disturbed sleep is connected to elevated cancer risk (Lehrer et al., 2013).

**FIG. 4.**
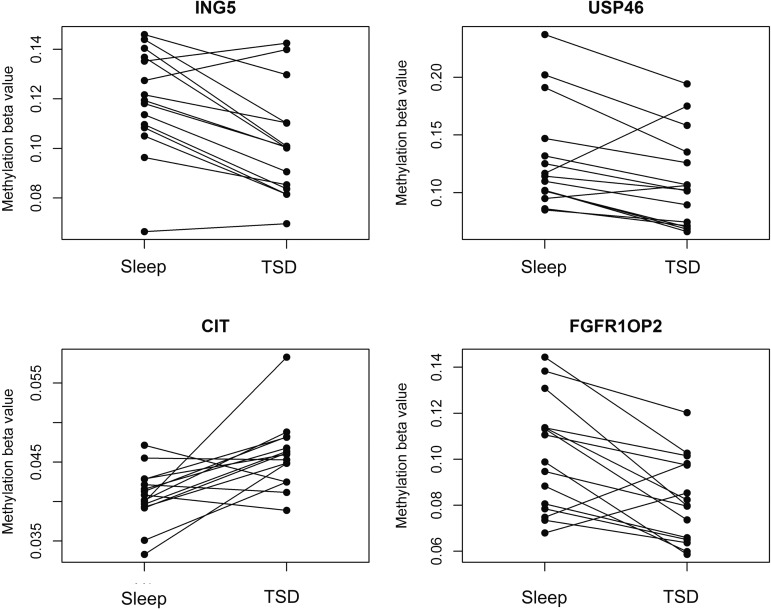
Targeted analysis of genes known to be deferentially expressed in sleep deprivation: Targeted analysis of TSD-dependent methylation changes in genes reported to be differentially expressed in TSD by Möller-Levet et al. (E-GEOD-48113). Shown are ladder plots of the four probes that passed both steps I (paired *t*-test *p* value <10^−2.5^) and II analyses (methylation change larger than the median change in technical replicates after Benjamini–Hochberg correction). With the exception of the probe associated with *ING5*, all CpG sites were located downstream of the respective transcription start site (USP46/cg25019777 99 bp, CIT/cg06887167 416 bp, and FGFR1OP2/cg17643699 180 bp).

A targeted analysis revealed three additional genes (*CIT*, *USP46*, and *FGFR1OP2*) showing both differential methylation in TSD and time awake-dependent changes in expression in humans. All three genes were transcriptionally repressed, and methylation data indicated increased methylation in *CIT* and decreased methylation in *USP56* and *FGFR1OP2* (Fig. 4). In animal experiments, *USP46* has been shown to be responsible for the induction symptoms of depression, as measured by the tail mobility in a CS mouse strain (Cryan and Mombereau, 2004). SNP analyses in a Japanese population confirmed *USP46* as a gene linked to major depressive disorder in humans (Fukuo et al., 2011). These observations allow the hypothesis that TSD has the ability to induce a shift in *USP46* gene expression through the induction of methylation changes, which may be at least partly responsible for a higher susceptibility to depressive symptoms as shown, especially for longer periods of sleep deprivation in humans (Banks and Dinges, 2007; Pizzagalli et al., 2001).

We detected two specific signaling pathways to be epigenetically affected by TSD, that is, the Notch and Wnt signaling pathways. Notch signaling has been associated with embryonic development and cell fate (Artavanis-Tsakonas et al., 1999), and disruptions in the signaling pathway possibly lead to various cancers. With disruptions in the cell fate determining mechanisms possibly leading to various cancers. The Wnt pathway plays a role in cancerogenesis as well. Stem cell cancer growth can be stopped by inhibiting key features of Notch and Wnt signaling (Takebe et al., 2011). Both pathways are also involved in stress response. Thus, it cannot be entirely discriminated whether the methylation changes observed in these pathways are a result of a stress response induced by one night TSD or a direct consequence of TSD.

Furthermore, the Wnt signaling pathway has been demonstrated to have implications in the pathogenesis of bipolar disorder, which is partly characterized by sleep disturbances. Interestingly, changes in the functionality of both Notch and Wnt signaling have been linked to changes in the secretion of cortisol. In addition to these findings, glucocorticoids have been demonstrated to mediate hematopoietic stem and progenitor cell function through Notch1 signaling (Kollet et al., 2013) and to suppress the canonical Wnt signaling pathways in cultured human osteoblasts (Ohnaka et al., 2005). Thus, our findings support the hypothesis of an epigenetic link between sleep deprivation and depression through modulations of the Wnt and Notch signaling pathways.

We predicted that the heat shock protein 4 (HSPA4) has a high potential to physically interact with other genes as given in Supplementary Table S1. We furthermore showed that *HSPA4* is part of a methylation–methylation correlation cluster together with six other genes (*MYO5A, BC040577 GALNT7, KREMEN1, MICAL3, CECR6,* and *GPR155*). Heat shock proteins respond to various external stressors, one of which is sleep deprivation (Ackermann et al., 2013). The demethylation of the promoter region of *HSPA4* observed in this study could be a compensatory response to promote sleep in our subjects (Ekimova, 2013). The casein kinases CSNK2A1, which has been shown to be connected to the WNT signaling pathway (Gao and Wang, 2006), and CSNK1G2, which induces proteasomal degradation of the period circadian clock 1 protein (PER1) (Lee et al., 2009), are predicted to physically interact with HSPA4. On the basis of our observations, we speculate that induced demethylation and subsequent overexpression of CSNK2A1 may lead to a functional impairment of PER1 function by means of degradation.

Because TSD is able to induce significant shifts in the N/L ratio, we performed our investigations by taking the N/L ratio into account since especially long-term effects of sleep deprivation on white blood cell subpopulations have been described (Lasselin et al., 2014). The results of this and other studies indicate a bidirectional association between sleep, the immune system, and inflammatory markers (Christoffersson et al., 2014). Based on the obtained results, we hypothesize that changes in the N/L ratio are reflected in shifts in the methylation profile that are disproportionately located in LC regions. Interestingly and in contrast to that, the N/L ratio-independent methylation changes were primarily located in HC regions. This observation suggests that HC probes are less sensitive to shifts in white blood cell composition, thus, reflecting induced changes in DNA methylation of likely functional relevance in a better way. In summary, this study demonstrates that TSD is able to induce significant changes in the genome-wide methylation profile with potential consequences for the functionality of regulatory pathways involved in Notch and Wnt signaling, *ING5* and *HSPA4* regulation, and that TSD-induced shifts in monocyte subpopulation are most prominent in LC islands.

### Study limitations

Our study was performed on whole blood samples. It cannot be excluded that changes in the methylation pattern is reflective of the tissue used. Recent studies, though, show that the DNA methylation profile shows a strong overlap between the blood and brain (Horvath et al., 2012), which made it reasonable to perform our studies on whole blood. At this point, it is uncertain to which extent the reported changes are reversible or if they persist. The potential for long-term conclusions from this article is limited. A majority of the differentially methylated sites were correlated with the N/L ratio (Fig. 2a), indicating an effect of sleep deprivation on immune processes. However, by measuring and adjusting for the N/L ratio allowed us to identify DNA methylation changes that were independent of this effect.

It would be of value that future studies focus on studying methylation changes in different tissue types. The methylation data were compared with expression data of subjects of comparable age (mean = 27.5, SD = 4.3), but there were demographic differences. Möller-Levet et al. included women (12 of 26) and individuals with a different ethnical background compared with those in our study (7 of 26 were nonwhite). Moreover, Möller-Levet et al. studied the effect of time awake as opposed to acute sleep deprivation, which partly impedes the direct comparability of the study outcomes.

The homogeneous composition of the subject group used in this study has both strengths and limitations. Although it reduces the influence of TSD-unrelated variance, it limits the extent to which the data can be extrapolated. Gender, ethnicity, age, and obesity are just some of the factors that are known to influence both DNA methylation and sleep patterns.

## Supplementary Material

Supplemental data

## Abbreviations Used

N/L: neutrophil/leukocyte
TSD: total sleep deprivation
TSS: transcription start site

## References

[B1] Ackermann K, Plomp R, Lao O, et al. (2013). Effect of sleep deprivation on rhythms of clock gene expression and melatonin in humans. Chronobiol Int 30, 901–909 23738906 10.3109/07420528.2013.784773

[B2] Artavanis-Tsakonas S, Rand MD, and Lake RJ (1999). Notch signaling: Cell fate control and signal integration in development. Science 284, 770–776 10221902 10.1126/science.284.5415.770

[B3] Banks S, and Dinges DF (2007). Behavioral and physiological consequences of sleep restriction. J Clin Sleep Med 3, 519–528 17803017 PMC1978335

[B4] Benedict C, Cedernaes J, Giedraitis V, et al. (2014). Acute sleep deprivation increases serum levels of neuron-specific enolase (NSE) and S100 calcium binding protein B (S-100B) in healthy young men. Sleep 37, 195–198 24470708 10.5665/sleep.3336 PMC3902870

[B5] Buhule OD, Minster RL, Hawley NL, et al. (2014). Stratified randomization controls better for batch effects in 450K methylation analysis: a cautionary tale. Front Genet 5, 354 25352862 10.3389/fgene.2014.00354 PMC4195366

[B6] Cedernaes J, Osler ME, Voisin S, et al. (2015). Acute sleep loss induces tissue-specific epigenetic and transcriptional alterations to Circadian clock genes in men. J Clin Endocrinol Metab 100, E1255–61 26168277 10.1210/JC.2015-2284

[B7] Christoffersson G, Vågesjö E, Pettersson US, et al. (2014). Acute sleep deprivation in healthy young men: Impact on population diversity and function of circulating neutrophils. Brain Behav Immun 41, 162–172 24878171 10.1016/j.bbi.2014.05.010

[B8] Cryan JF, and Mombereau C (2004). In search of a depressed mouse: utility of models for studying depression-related behavior in genetically modified mice. Mol Psychiatry 9, 326–357 14743184 10.1038/sj.mp.4001457

[B9] Doi M, Hirayama J, and Sassone-Corsi P (2006). Circadian regulator CLOCK is a histone acetyltransferase. Cell 125, 497–508 16678094 10.1016/j.cell.2006.03.033

[B10] Du P, Zhang X, Huang C-C, et al. (2010). Comparison of Beta-value and M-value methods for quantifying methylation levels by microarray analysis. BMC Bioinformatics 11, 587 21118553 10.1186/1471-2105-11-587 PMC3012676

[B11] Ekimova IV (2013). Somnogenic effect of exogenous heat shock protein 70 kDa is mediated by GABA(A) receptors in the preoptic area of the hypothalamus. Dokl Biol Sci 449, 89–92 23652435 10.1134/S0012496613020130

[B12] Fouse SD, Shen Y, Pellegrini M, et al. (2008). Promoter CpG methylation contributes to ES cell gene regulation in parallel with Oct4/Nanog, PcG complex, and histone H3 K4/K27 trimethylation. Cell Stem Cell 2, 160–169 18371437 10.1016/j.stem.2007.12.011 PMC3070208

[B13] Fukuo Y, Kishi T, Kushima I, et al. (2011). Possible association between ubiquitin-specific peptidase 46 gene and major depressive disorders in the Japanese population. J Affect Disord 133, 150–157 21663972 10.1016/j.jad.2011.04.020

[B14] Gao Y, and Wang H-Y (2006). Casein kinase 2 is activated and essential for Wnt/β-catenin signaling. J Biol Chem 281, 18394–18400 16672224 10.1074/jbc.M601112200

[B15] Gentleman R, Carey V, Bates D, et al. (2004). Bioconductor: open software development for computational biology and bioinformatics. Genome Biol 5, R80 15461798 10.1186/gb-2004-5-10-r80 PMC545600

[B16] Horvath S, Zhang Y, Langfelder P, et al. (2012). Aging effects on DNA methylation modules in human brain and blood tissue. Genome Biol 13, R97 23034122 10.1186/gb-2012-13-10-r97 PMC4053733

[B17] Irwin MR, Wang M, Campomayor CO, Collado-Hidalgo A, and Cole S (2006). SLeep deprivation and activation of morning levels of cellular and genomic markers of inflammation. Arch Intern Med 166, 1756–1762 16983055 10.1001/archinte.166.16.1756

[B18] Kamburov A, Pentchev K, Galicka H, Wierling C, Lehrach H, and Herwig R (2011). ConsensusPathDB: toward a more complete picture of cell biology. Nucleic Acids Res 39, D712–D717 21071422 10.1093/nar/gkq1156 PMC3013724

[B19] Kollet O, Vagima Y, D'uva G, et al. (2013). Physiologic corticosterone oscillations regulate murine hematopoietic stem/progenitor cell proliferation and CXCL12 expression by bone marrow stromal progenitors. Leukemia 27, 2006–2015 23680895 10.1038/leu.2013.154

[B20] Lasselin J, Rehman J-U, Åkerstedt T, Lekander M, and Axelsson J (2015). Effect of long-term sleep restriction and subsequent recovery sleep on the diurnal rhythms of white blood cell subpopulations. Brain Behav Immun 47, 93–99 25451611 10.1016/j.bbi.2014.10.004

[B21] Lee H, Chen R, Lee Y, Yoo S, and Lee C (2009). Essential roles of CKIδ and CKIɛ in the mammalian circadian clock. Proc Natl Acad Sci U S A 106, 21359–21364 19948962 10.1073/pnas.0906651106 PMC2795500

[B22] Lehrer S, Green S, Ramanathan L, and Rosenzweig KE (2013). Obesity and deranged sleep are independently associated with increased cancer mortality in 50 US states and the District of Columbia. Sleep Breath 17, 1117–1118 23389836 10.1007/s11325-013-0811-x

[B23] Liu N, Wang J, Wang J, et al. (2013). ING5 is a Tip60 cofactor that acetylates p53 in response to DNA damage. Cancer Res 73, 3749–3760 23576563 10.1158/0008-5472.CAN-12-3684

[B24] Massart R, Freyburger M, Suderman M, et al. (2014). The genome-wide landscape of DNA methylation and hydroxymethylation in response to sleep deprivation impacts on synaptic plasticity genes. Transl Psychiatry 4, e347 24448209 10.1038/tp.2013.120 PMC3905230

[B25] Möller-Levet CS, Archer SN, Bucca G, et al., (2013). Effects of insufficient sleep on circadian rhythmicity and expression amplitude of the human blood transcriptome. Proc Natl Acad Sci U S A 110, E1132–E1141 23440187 10.1073/pnas.1217154110 PMC3607048

[B26] Mostafavi S, Ray D, Warde-Farley D, Grouios C, and Morris Q (2008). GeneMANIA: a real-time multiple association network integration algorithm for predicting gene function. Genome Biol 9 Suppl 1, S4 18613948 10.1186/gb-2008-9-s1-s4 PMC2447538

[B27] Ohnaka K, Tanabe M, Kawate H, Nawata H, and Takayanagi R (2005). Glucocorticoid suppresses the canonical Wnt signal in cultured human osteoblasts. Biochem Biophys Res Commun 329, 177–181 15721290 10.1016/j.bbrc.2005.01.117

[B28] Pico AR, Kelder T, Van Iersel MP, Hanspers K, Conklin BR, and Evelo C, (2008). WikiPathways: pathway editing for the people. PLoS Biol 6, e184 18651794 10.1371/journal.pbio.0060184 PMC2475545

[B29] Pizzagalli D, Pascual-Marqui RD, Nitschke JB, et al. (2001). Anterior cingulate activity as a predictor of degree of treatment response in major depression: Evidence from brain electrical tomography analysis. Am J Psychiatry 158, 405–415 11229981 10.1176/appi.ajp.158.3.405

[B30] Price ME, Cotton AM, Lam LL, et al. (2013). Additional annotation enhances potential for biologically-relevant analysis of the Illumina Infinium HumanMethylation450 BeadChip array. Epigenetics Chromatin 6, 4 23452981 10.1186/1756-8935-6-4 PMC3740789

[B31] Qureshi IA, and Mehler MF (2014). Epigenetics of sleep and chronobiology. Curr Neurol Neurosci 14, 432 10.1007/s11910-013-0432-6 PMC3957188 24477387

[B32] Smyth GK (2005). Limma: Linear Models for Microarray Data. New York, NY: Springer

[B33] Steegenga W, Boekschoten M, Lute C, et al. (2014). Genome-wide age-related changes in DNA methylation and gene expression in human PBMCs. AGE 36, 1523–1540 10.1007/s11357-014-9648-x PMC4082572 24789080

[B34] Takebe N, Harris PJ, Warren RQ, and Ivy SP (2011). Targeting cancer stem cells by inhibiting Wnt, Notch, and Hedgehog pathways. Nat Rev Clin Oncol 8, 97–106 21151206 10.1038/nrclinonc.2010.196

[B35] Team RC (2011). R: A Language and Environment for Statistical Computing. Vienna, Austria: The R Foundation for Statistical Computing ISBN:3-900051-07-0

[B36] Teschendorff AE, Marabita F, Lechner M, et al. (2013). A beta-mixture quantile normalization method for correcting probe design bias in Illumina Infinium 450 k DNA methylation data. Bioinformatics 29, 189–196 23175756 10.1093/bioinformatics/bts680 PMC3546795

[B37] Wagner JR, Busche S, Ge B, Kwan T, Pastinen T, and Blanchette M (2014). The relationship between DNA methylation, genetic and expression inter-individual variation in untransformed human fibroblasts. Genome Biol 15, R37 24555846 10.1186/gb-2014-15-2-r37 PMC4053980

[B38] Xing YN, Yang X, Xu XY, et al. (2011). The altered expression of ING5 protein is involved in gastric carcinogenesis and subsequent progression. Hum Pathol 42, 25–35 21062663 10.1016/j.humpath.2010.05.024

[B39] Yousefi P, Huen K, Schall RA, et al. (2013). Considerations for normalization of DNA methylation data by Illumina 450K BeadChip assay in population studies. Epigenetics 8, 1141–1152 23959097 10.4161/epi.26037 PMC6242262

[B40] Zheng HC, Xia P, Xu XY, Takahashi H, and Takano Y, (2011). The nuclear to cytoplasmic shift of ING5 protein during colorectal carcinogenesis with their distinct links to pathologic behaviors of carcinomas. Hum Pathol 42, 424–433 21193223 10.1016/j.humpath.2009.12.018

