# Supplementary material for: Epigenomics of Total Acute Sleep Deprivation in Relation to Genome-Wide DNA Methylation Profiles and RNA Expression
Source: OMICS. 2016 Jun 1;20(6):334–42. doi: 10.1089/omi.2016.0041 (PMC4926204; doi:10.1089/omi.2016.0041)
Supplement: Supplemental data [file Supp_Table1.docx]

**Supplementary table 1:** Descriptive information about the 269 differentially methylated probes.

| **Closest gene name and ID** | **Distance to TSS** | **Step I P-value** | **Step II P-value** | **Sleep mean (sd)** | **Wake mean (sd)** | **Number hits/tot** | **Correlation coef.(P)** |
| --- | --- | --- | --- | --- | --- | --- | --- |
| UBR7 cg01261464 | -225 | 1.96E-05 | 1.07E-04 | 4.28 (1.27) | 3.1 (0.34) | 1/6 | -0.15 (0.34) |
| C11orf91 cg04023915 | 224 | 2.16E-05 | 3.80E-03 | 16.77 (2.83) | 13.82 (1.66) | 3/11 | N/A |
| EEF1E1 cg07345919 | -91 | 2.31E-05 | 2.72E-03 | 8.46 (1.75) | 6.33 (0.88) | 1/9 | -0.07 (0.43) |
| AK097012 cg26498966 | 1793 | 3.05E-05 | 4.95E-03 | 96.02 (1.17) | 97.11 (0.57) | 1/4 | N/A |
| FZD6 cg24115571 | 53 | 3.09E-05 | 1.28E-03 | 9.61 (1.99) | 12.04 (0.41) | 1/9 | 0.55 (0.05)^a^ |
| ING5 cg03000844 | -69 | 6.87E-05 | 5.76E-04 | 11.92 (2.1) | 10.18 (0.92) | 2/8 | 0.28 (0.22) |
| KREMEN1 cg20742366 | -68 | 7.38E-05 | 1.63E-06 | 13.9 (2.62) | 11.24 (0.51) | 2/12 | -0.19 (0.3) |
| HPGD cg13181537 | -76 | 7.48E-05 | 6.59E-03 | 9.63 (1.24) | 11.65 (1.04) | 1/7 | 0.41 (0.12) |
| GLCCI1 cg19438100 | -1 | 7.93E-05 | 1.38E-03 | 8.53 (2.06) | 6.42 (0.59) | 1/8 | -0.45 (0.09) |
| RGS12 cg18230796 | -162 | 8.74E-05 | 3.66E-03 | 7.33 (1.09) | 5.82 (0.75) | 2/53 | -0.15 (0.34) |
| VPS37C cg02731980 | -303 | 9.46E-05 | 7.71E-03 | 7.73 (1.07) | 6.39 (0.83) | 2/14 | 0.31 (0.19) |
| ZNF37A cg17272795 | -414 | 1.02E-04 | 1.59E-03 | 9.43 (1.93) | 9.86 (0.55) | 1/1 | 0.12 (0.37) |
| TADA2A cg01929530 | 96 | 1.24E-04 | 2.56E-05 | 9.58 (2.19) | 7.34 (0.43) | 1/6 | -0.62 (0.03) ^a^ |
| PHOX2A cg13103303 | 107 | 1.29E-04 | 1.45E-02 | 3.53 (0.69) | 2.63 (0.51) | 1/15 | -0.44 (0.1) |
| DNAJC3 cg14806867 | -37 | 1.33E-04 | 9.82E-03 | 18.36 (2.37) | 15.86 (1.33) | 4/7 | 0.61 (0.03) ^a^ |
| DHRS12 cg16948199 | 468 | 1.54E-04 | 1.56E-04 | 2.03 (0.46) | 1.69 (0.07) | 1/13 | 0.13 (0.36) |
| CECR6 cg03282993 | -2 | 1.67E-04 | 6.45E-03 | 18.02 (3.2) | 14.49 (1.63) | 1/6 | -0.07 (0.42) |
| UBE4B cg09355820 | -803 | 1.82E-04 | 3.94E-04 | 86.42 (1.31) | 88.77 (0.98) | 1/12 | 0.36 (0.15) |
| MAT2A cg19276031 | -253 | 1.95E-04 | 7.24E-04 | 5.59 (0.62) | 6.29 (0.34) | 1/7 | -0.38 (0.14) |
| CSNK2A1 cg19292062 | 138 | 2.00E-04 | 6.71E-03 | 13.22 (1.44) | 11.48 (0.94) | 1/16 | -0.11 (0.38) |
| C8orf33 cg14844679 | 177 | 2.08E-04 | 6.11E-03 | 7.51 (1.11) | 9.35 (1.28) | 1/11 | -0.48 (0.08) |
| HOXC6 cg22378817 | 295 | 2.12E-04 | 2.27E-03 | 12.02 (1.41) | 10.94 (0.68) | 1/6 | 0.38 (0.13) |
| C1orf173 cg15386773 | -68 | 2.18E-04 | 4.49E-03 | 16.15 (4.12) | 13.68 (0.91) | 1/12 | 0.29 (0.20) |
| JTB  cg06324094 | -31 | 2.19E-04 | 4.73E-04 | 14.77 (2.81) | 12.08 (0.77) | 1/12 | 0.28 (0.22) |
| SOX1 cg25463470 | -200 | 2.21E-04 | 1.36E-02 | 3.2 (0.94) | 3.7 (0.35) | 1/20 | -0.29 (0.20) |
| RDH13 cg02279127 | -117 | 2.26E-04 | 5.77E-04 | 9.98 (2.35) | 7.57 (1.24) | 2/7 | -0.34 (0.16) |
| PMEL cg16190688 | 636 | 2.30E-04 | 3.94E-05 | 88.16 (2.02) | 90.13 (0.8) | 1/8 | N/A |
| SEL1L2 cg04521027 | 198 | 2.36E-04 | 4.59E-03 | 89.12 (2.33) | 87.16 (2.02) | 2/6 | 0.01 (0.49) |
| NETO1 cg23505303 | 700 | 2.39E-04 | 1.79E-03 | 8.88 (1.89) | 9.55 (0.74) | 1/9 | -0.16 (0.33) |
| ZNF678 cg26683023 | -327 | 2.56E-04 | 1.51E-03 | 86.46 (1.57) | 88.13 (0.51) | 1/12 | -0.21 (0.28) |
| RPL26L1 cg01418033 | 103 | 2.65E-04 | 4.72E-03 | 7.02 (1.25) | 6.45 (0.71) | 1/8 | 0.10 (0.39) |
| ZNF354A cg26433777 | 357 | 2.68E-04 | 9.24E-07 | 11.78 (2.09) | 10.32 (0.28) | 1/3 | -0.54 (0.05) ^a^ |
| CSNK1G2 cg08184586 | -743 | 2.82E-04 | 3.15E-03 | 7.9 (1.3) | 6.55 (0.94) | 1/15 | -0.24 (0.25) |
| ODC1 cg14276772 | -193 | 2.91E-04 | 1.18E-02 | 6.67 (1.43) | 5.39 (0.82) | 2/10 | 0.33 (0.17) |
| MBOAT2 cg14909930 | -118 | 2.98E-04 | 1.49E-03 | 18.33 (3.63) | 14.91 (1.73) | 4/14 | -0.01 (0.50) |
| SPEG cg03030650 | 1981 | 3.06E-04 | 4.04E-04 | 95.87 (0.72) | 96.56 (0.35) | 2/37 | -0.46 (0.09) |
| VOPP1 cg05369274 | -14 | 3.15E-04 | 2.02E-04 | 8.58 (1.3) | 7.05 (0.73) | 1/17 | -0.03 (0.47) |
| FRAT1 cg15432938 | -209 | 3.18E-04 | 1.72E-02 | 5.42 (0.46) | 6.15 (0.42) | 1/11 | -0.57 (0.04) ^a^ |
| RCOR1 cg08707819 | 396 | 3.33E-04 | 9.60E-04 | 44.85 (3.41) | 40.67 (1.5) | 1/10 | -0.33 (0.17) |
| FEM1C cg21199023 | -154 | 3.50E-04 | 5.79E-03 | 4.6 (0.97) | 3.53 (0.55) | 3/11 | -0.29 (0.21) |
| CYR61 cg25720697 | -66 | 3.59E-04 | 1.30E-03 | 7.03 (0.87) | 8.13 (0.58) | 3/7 | 0.06 (0.43) |
| CRNDE cg07844687 | -165 | 3.62E-04 | 6.14E-03 | 8.31 (1.48) | 6.79 (1.02) | 1/10 | N/A |
| PRUNE2 cg14489366 | -79 | 3.64E-04 | 9.61E-05 | 14.51 (2.24) | 12.96 (0.06) | 1/8 | 0.01 (0.50) |
| PITPNM3 cg12473340 | -1297 | 3.69E-04 | 8.10E-03 | 93.67 (1.26) | 92.46 (0.72) | 4/12 | 0.03 (0.46) |
| PARVB cg11553066 | 601 | 3.80E-04 | 1.53E-03 | 14.87 (2.47) | 17.66 (1.05) | 1/9 | 0.71 (0.01) ^a^ |
| DPH1 cg02340576 | 296 | 4.00E-04 | 1.35E-02 | 4.25 (0.68) | 4.83 (0.42) | 1/12 | -0.26 (0.23) |
| ACSL3 cg19738182 | 458 | 4.03E-04 | 6.81E-03 | 7.27 (1.31) | 5.87 (0.98) | 1/8 | 0.53 (0.06) |
| ZC3H10 cg16134607 | 62 | 4.50E-04 | 2.26E-04 | 19.26 (3.76) | 16.43 (1.4) | 2/11 | 0.05 (0.44) |
| C3orf70 cg18082112 | 665 | 4.55E-04 | 2.61E-03 | 15.08 (2.54) | 14.17 (1) | 1/11 | 0.44 (0.10) |
| C16orf89 cg02768760 | -1678 | 4.57E-04 | 4.51E-03 | 79.32 (3.67) | 82.01 (2.41) | 1/4 | 0.04 (0.45) |
| FAM126B cg02994018 | 291 | 4.80E-04 | 8.53E-03 | 11 (2.63) | 9.16 (1.03) | 3/7 | 0.58 (0.04) ^a^ |
| ZNF642 cg22449337 | 612 | 4.93E-04 | 7.63E-04 | 89.04 (2.1) | 90.41 (1.08) | 3/7 | -0.17 (0.32) |
| KIF6 cg14245120 | -563 | 5.07E-04 | 2.14E-04 | 85.22 (2.09) | 87.65 (0.66) | 2/18 | -0.43 (0.11) |
| AX747860 cg06296503 | 761 | 5.12E-04 | 5.69E-03 | 8.82 (1.37) | 7.67 (0.47) | 1/8 | N/A |
| GAB1 cg14953026 | -297 | 5.14E-04 | 1.41E-02 | 5.79 (1.1) | 4.6 (0.81) | 1/12 | 0.26 (0.23) |
| OR11G2 cg13183731 | -914 | 5.16E-04 | 1.03E-03 | 87.15 (3.9) | 84.51 (1.32) | 2/2 | -0.30 (0.20) |
| KLF3 cg18192146 | -185 | 5.26E-04 | 1.49E-02 | 7.58 (1.35) | 6.22 (0.84) | 1/7 | -0.46 (0.09) |
| EXOSC6 cg27468401 | 425 | 5.51E-04 | 3.64E-03 | 7.19 (1.23) | 6.28 (0.3) | 3/11 | 0 (0.5) |
| TMEM158 cg08483723 | -146 | 5.59E-04 | 2.80E-04 | 16.16 (3.25) | 13.14 (1.14) | 2/7 | -0.74 (0.01) ^a^ |
| SOX7 cg14855657 | 418 | 5.75E-04 | 1.94E-02 | 9.1 (1.37) | 7.91 (1.44) | 2/7 | 0.15 (0.34) |
| AX746710 cg08700032 | 78 | 5.77E-04 | 2.17E-03 | 8.75 (1.04) | 7.58 (0.98) | 1/1 | N/A |
| LOC646762 cg00313795 | 241 | 5.81E-04 | 1.76E-02 | 7.19 (1.78) | 5.6 (0.99) | 1/3 | N/A |
| EMX1 cg08013270 | 1141 | 5.92E-04 | 5.82E-05 | 12.38 (1.03) | 13.96 (0.11) | 1/15 | -0.66 (0.02) ^a^ |
| NISCH cg04400147 | 1035 | 6.02E-04 | 1.57E-03 | 98.07 (0.45) | 98.52 (0.11) | 1/14 | -0.05 (0.45) |
| PSMB10 cg06655217 | -85 | 6.06E-04 | 1.19E-03 | 6.34 (0.52) | 7.4 (0.54) | 2/12 | -0.16 (0.32) |
| FAM43B cg20404387 | 365 | 6.10E-04 | 1.33E-02 | 10.23 (1.38) | 11.99 (1.25) | 3/14 | 0.01 (0.49) |
| PBMUCL2 cg17038049 | 139 | 6.28E-04 | 1.64E-03 | 87.06 (1.89) | 85.4 (0.71) | 2/3 | N/A |
| PAN2 cg09109477 | -24 | 6.45E-04 | 9.39E-03 | 5.21 (0.67) | 5.97 (0.33) | 3/14 | 0.30 (0.20) |
| ZBED5 cg13499067 | -9 | 6.56E-04 | 4.18E-03 | 8.63 (1.03) | 7.54 (0.51) | 1/5 | -0.01 (0.49) |
| ZCRB1 cg01182690 | 388 | 6.70E-04 | 8.31E-04 | 8.9 (1.39) | 7.31 (0.77) | 3/5 | -0.34 (0.17) |
| ABCD2 cg08424219 | -218 | 6.72E-04 | 1.48E-02 | 8.15 (1.09) | 9.53 (0.76) | 1/4 | 0.01 (0.50) |
| TFF3 cg20488657 | -1220 | 6.80E-04 | 7.18E-03 | 85.95 (1.13) | 84.69 (0.8) | 1/5 | -0.15 (0.34) |
| CPAMD8 cg17409891 | -60 | 7.02E-04 | 1.75E-03 | 10.83 (1.77) | 8.75 (0.72) | 3/16 | -0.33 (0.17) |
| AX747817 cg27601574 | 1002 | 7.29E-04 | 3.71E-03 | 89.2 (1.5) | 87.66 (1.29) | 2/4 | N/A |
| SNORD115-37 cg17208360 | -44 | 7.39E-04 | 1.38E-03 | 74.5 (2.01) | 73.06 (0.78) | 1/3 | 0.37 (0.14) |
| FAT1 cg05321808 | 393 | 7.42E-04 | 2.36E-03 | 11.41 (1.9) | 9.04 (0.66) | 1/21 | -0.29 (0.21) |
| OR52I2 cg03067903 | -1388 | 7.48E-04 | 3.87E-04 | 92.09 (1.21) | 91 (0.55) | 1/1 | -0.03 (0.47) |
| WNT4 cg20792512 | 329 | 7.62E-04 | 3.23E-03 | 1.21 (0.25) | 0.94 (0.24) | 1/7 | 0.5 (0.07) |
| NDUFS7 cg07652592 | 96 | 7.67E-04 | 4.87E-03 | 3.62 (0.63) | 3.03 (0.37) | 1/8 | -0.05 (0.45) |
| MRPS5 cg25820224 | 203 | 7.69E-04 | 2.75E-04 | 12.38 (1.86) | 10.31 (0.8) | 1/12 | 0.09 (0.40) |
| CLUAP1 cg02073511 | -38 | 7.71E-04 | 1.30E-03 | 11.78 (1.95) | 10.12 (0.84) | 2/10 | -0.02 (0.47) |
| HES1 cg24182293 | -932 | 7.75E-04 | 8.02E-03 | 8.9 (1.31) | 7.55 (0.57) | 1/28 | -0.12 (0.37) |
| FAM194B cg05399692 | -324 | 7.86E-04 | 2.12E-03 | 73.65 (2.89) | 71.63 (0.76) | 1/8 | -0.42 (0.11) |
| MOAP1 cg05199950 | 80 | 7.92E-04 | 2.77E-03 | 3.25 (0.64) | 2.54 (0.32) | 1/7 | 0.31 (0.19) |
| TMEM242 cg24931561 | -129 | 8.23E-04 | 1.94E-02 | 11.74 (2.31) | 10.75 (1.23) | 1/11 | N/A |
| FSIP1 cg22936016 | -148 | 8.83E-04 | 3.58E-03 | 8.36 (2.27) | 6.59 (1.02) | 4/13 | -0.73 (0.01) ^a^ |
| ALX4 cg26717041 | -1038 | 8.88E-04 | 1.16E-03 | 15.18 (1.82) | 16.9 (0.99) | 4/35 | -0.46 (0.09) |
| met-TRNA cg07588439 | 1527 | 8.90E-04 | 2.16E-04 | 75.45 (3.46) | 76.31 (0.51) | 1/11 | 0.51 (0.06) |
| KCNA10 cg03984537 | -1440 | 8.94E-04 | 1.71E-03 | 93.67 (1) | 93.14 (0.47) | 2/7 | -0.2 (0.29) |
| MYO1D cg16047348 | -329 | 9.05E-04 | 2.22E-05 | 8.27 (1.22) | 7.18 (0.14) | 1/6 | 0.29 (0.21) |
| YWHAZ cg06692871 | 224 | 9.12E-04 | 2.06E-04 | 1.63 (0.43) | 1.93 (0.14) | 2/20 | 0.19 (0.30) |
| EXOG cg24069376 | -182 | 9.21E-04 | 2.15E-03 | 27.56 (4.69) | 25.62 (1.6) | 1/5 | -0.56 (0.04) ^a^ |
| KBTBD4 cg02407032 | 70 | 9.32E-04 | 1.14E-02 | 13.18 (2.52) | 10.56 (1.58) | 3/16 | 0.17 (0.32) |
| MYO1C cg08051602 | 298 | 9.34E-04 | 1.55E-02 | 10.31 (1.96) | 7.83 (1.39) | 2/19 | 0.08 (0.41) |
| MGP cg22221831 | -544 | 9.49E-04 | 1.16E-03 | 63.41 (7) | 67.21 (1.12) | 1/2 | -0.3 (0.20) |
| ZAP70 cg23963397 | -217 | 9.52E-04 | 1.15E-02 | 90.66 (1.16) | 89.53 (0.84) | 3/30 | -0.06 (0.44) |
| CUL5 cg00047050 | 24 | 9.70E-04 | 9.19E-03 | 13.63 (2.04) | 15.66 (1.44) | 2/8 | -0.02 (0.47) |
| QTRT1 cg03247675 | 0 | 1.00E-03 | 1.70E-02 | 1.44 (0.45) | 1.75 (0.31) | 1/10 | -0.19 (0.30) |
| CYGB cg00958884 | -194 | 1.01E-03 | 6.13E-03 | 8.05 (1.51) | 6.5 (0.89) | 2/12 | 0.54 (0.05) ^a^ |
| INHBA cg16881064 | 529 | 1.02E-03 | 3.23E-05 | 8.85 (2.37) | 6.7 (0.7) | 2/11 | -0.04 (0.46) |
| UBR1 cg21590616 | 16 | 1.03E-03 | 1.49E-03 | 9.07 (1.5) | 7.44 (0.72) | 2/8 | -0.28 (0.21) |
| EIF4EBP1 cg04423064 | -93 | 1.03E-03 | 1.21E-02 | 6.01 (1.03) | 5.03 (0.66) | 2/13 | 0.51 (0.06) |
| FZD8 cg08495456 | -802 | 1.03E-03 | 5.13E-05 | 8.65 (2.08) | 6.95 (0.21) | 2/15 | 0.02 (0.48) |
| SSH3 cg25753024 | -5 | 1.04E-03 | 9.31E-03 | 11.84 (4.4) | 12.47 (1.65) | 2/12 | 0.27 (0.23) |
| TYW3 cg09502221 | -236 | 1.05E-03 | 2.29E-03 | 6.18 (3.61) | 5.04 (0.64) | 1/6 | 0.12 (0.37) |
| TRNA_Met cg26995992 | -92 | 1.06E-03 | 4.39E-03 | 6.18 (1.29) | 5.18 (0.47) | 6/54 | N/A |
| DMRTC2 cg07890457 | -201 | 1.07E-03 | 5.70E-03 | 92.7 (2.33) | 91.03 (1.06) | 1/4 | 0.57 (0.04) ^a^ |
| LHPP cg17946375 | -66 | 1.08E-03 | 4.74E-03 | 1.5 (0.39) | 1.78 (0.21) | 1/8 | -0.21 (0.28) |
| KIAA1841 cg17451422 | -109 | 1.08E-03 | 2.53E-03 | 12.79 (2.69) | 10.77 (0.98) | 2/14 | 0.24 (0.25) |
| NPBWR2 cg04137594 | 218 | 1.10E-03 | 1.12E-03 | 73.2 (1.57) | 71.47 (1.28) | 4/9 | 0.46 (0.09) |
| FAM65A cg00586537 | -10 | 1.11E-03 | 9.07E-03 | 8.84 (1.58) | 7.12 (1.21) | 1/13 | -0.12 (0.37) |
| UFM1 cg07571046 | 466 | 1.11E-03 | 4.40E-04 | 6.44 (0.98) | 6.84 (0.43) | 1/7 | 0.19 (0.3) |
| RAET1L cg01878724 | -253 | 1.11E-03 | 1.07E-02 | 10.78 (2.48) | 12.67 (1.52) | 3/6 | -0.32 (0.18) |
| GGN cg00038736 | 1534 | 1.13E-03 | 1.31E-02 | 63.4 (4.13) | 58.83 (1.77) | 2/9 | 0.47 (0.08) |
| NAB2 cg19311889 | 1117 | 1.18E-03 | 7.25E-03 | 7.61 (1.61) | 6.51 (0.83) | 2/15 | 0.17 (0.32) |
| ATP13A2 cg15465571 | 215 | 1.22E-03 | 1.15E-02 | 8.07 (1.84) | 6.63 (0.72) | 2/10 | -0.18 (0.31) |
| MYEF2 cg20603888 | -415 | 1.24E-03 | 7.93E-03 | 90.33 (1.86) | 91.7 (0.6) | 1/13 | -0.44 (0.10) |
| CEP72 cg18786969 | -32 | 1.26E-03 | 1.61E-02 | 7.26 (1.66) | 5.63 (0.99) | 1/11 | -0.42 (0.11) |
| CCDC138 cg23922134 | 320 | 1.26E-03 | 6.48E-03 | 30.19 (6.27) | 27.35 (1.12) | 1/10 | 0.08 (0.41) |
| TAF4 cg21065401 | 809 | 1.27E-03 | 8.47E-03 | 8.52 (1.93) | 6.63 (1.24) | 1/9 | -0.13 (0.36) |
| C2orf49 cg12102766 | 25 | 1.28E-03 | 2.09E-03 | 7.66 (1.29) | 6.55 (0.59) | 2/5 | 0 (0.5) |
| PNMAL2 cg24718465 | 51 | 1.31E-03 | 1.82E-03 | 28.13 (6.72) | 23.58 (1.92) | 3/8 | N/A |
| AF086125 cg05703096 | 60 | 1.37E-03 | 2.01E-04 | 29.9 (2.27) | 27.6 (0.65) | 1/2 | N/A |
| CAMSAP2 cg01796184 | -589 | 1.39E-03 | 6.75E-03 | 10.64 (1.32) | 12.43 (1.05) | 1/8 | N/A |
| ARMC10 cg18031021 | 745 | 1.40E-03 | 2.04E-03 | 9.63 (1.54) | 11.2 (0.8) | 1/5 | -0.26 (0.23) |
| LMBRD1 cg09103184 | -9 | 1.42E-03 | 1.41E-02 | 4.47 (2.13) | 5.74 (0.99) | 1/13 | -0.24 (0.25) |
| ANKRD37 cg11595794 | -745 | 1.44E-03 | 1.50E-05 | 5.29 (0.25) | 6.3 (0.13) | 1/9 | 0.23 (0.26) |
| COL4A2 cg22757824 | 556 | 1.45E-03 | 1.25E-03 | 11.98 (1.59) | 10.24 (0.84) | 3/9 | -0.1 (0.39) |
| DEF8 cg02061948 | 290 | 1.46E-03 | 5.58E-03 | 16.77 (3.56) | 14.33 (1.29) | 1/11 | 0.19 (0.3) |
| EMR3 cg07998554 | -46 | 1.48E-03 | 1.30E-02 | 9.44 (2.24) | 10.46 (0.89) | 2/6 | -0.16 (0.33) |
| ARHGEF19 cg11895451 | -186 | 1.50E-03 | 3.40E-03 | 11.15 (1.98) | 11.69 (0.27) | 2/11 | -0.02 (0.48) |
| UBA7 cg19381811 | -322 | 1.55E-03 | 9.20E-03 | 27.51 (4.2) | 30.55 (1.88) | 1/4 | 0.12 (0.37) |
| EFNB2 cg14363981 | 827 | 1.56E-03 | 1.19E-02 | 12.98 (2.08) | 11.29 (1.4) | 3/11 | 0.49 (0.07) |
| AKR1D1 cg25271841 | -1049 | 1.57E-03 | 1.05E-05 | 89.06 (1.39) | 87.88 (0.23) | 1/7 | 0.04 (0.45) |
| BC041855 cg12836085 | -729 | 1.57E-03 | 3.46E-03 | 18.08 (2.92) | 15.51 (1.18) | 2/4 | N/A |
| OR8D4 cg25762654 | -393 | 1.59E-03 | 4.57E-03 | 80.83 (1.7) | 78.92 (0.29) | 1/4 | -0.08 (0.41) |
| PCDHB3 cg23918315 | -1208 | 1.60E-03 | 5.52E-03 | 31.06 (4.36) | 26.67 (1.74) | 1/4 | 0.37 (0.14) |
| FADS6 cg06257708 | -88 | 1.61E-03 | 1.10E-02 | 5.57 (1.27) | 4.83 (0.53) | 2/7 | -0.58 (0.04) |
| BC040572 cg01528492 | 688 | 1.61E-03 | 7.54E-03 | 45.15 (3.32) | 48.87 (2.62) | 1/3 | N/A |
| BOC cg07873980 | -26 | 1.64E-03 | 2.00E-03 | 5.52 (1.08) | 4.49 (0.61) | 1/15 | 0.84 (<0.01) ^a^ |
| C3orf58 cg10116431 | 289 | 1.66E-03 | 2.39E-05 | 9.43 (1.75) | 7.95 (0.73) | 3/19 | 0.69 (0.01) ^a^ |
| LOC286189 cg11137615 | -617 | 1.66E-03 | 2.78E-03 | 6.98 (1.17) | 8.4 (0.82) | 1/10 | N/A |
| CPLX2 cg26121591 | 64 | 1.66E-03 | 1.70E-02 | 8.93 (2.38) | 6.86 (0.97) | 1/17 | -0.1 (0.40) |
| CLIP4 cg03135351 | -49 | 1.67E-03 | 1.70E-02 | 3.16 (0.51) | 2.53 (0.36) | 2/16 | -0.3 (0.20) |
| ASXL1 cg21151769 | 330 | 1.67E-03 | 7.78E-03 | 15.39 (2.8) | 13.23 (0.82) | 2/10 | -0.01 (0.48) |
| PTS cg15594528 | -218 | 1.68E-03 | 5.07E-04 | 2.69 (0.37) | 2.96 (0.16) | 2/12 | 0.28 (0.21) |
| F2RL1 cg23141632 | -261 | 1.68E-03 | 4.81E-04 | 5.68 (0.92) | 5.12 (0.3) | 1/11 | -0.62 (0.03) ^a^ |
| MICAL3 cg05367846 | -195 | 1.70E-03 | 4.18E-04 | 33.04 (6.86) | 27.3 (1.03) | 2/19 | -0.06 (0.43) |
| MED6 cg04843851 | -31 | 1.70E-03 | 1.14E-02 | 8.4 (1.27) | 7.08 (0.91) | 2/9 | -0.7 (0.01 ) ^a^ |
| TCF19 cg00160818 | 54 | 1.72E-03 | 1.31E-03 | 10.88 (1.8) | 9.33 (0.67) | 1/19 | -0.01 (0.49) |
| GMPS cg01634119 | -314 | 1.73E-03 | 1.12E-02 | 6.01 (0.75) | 7.09 (0.63) | 1/12 | 0.32 (0.18) |
| PSMA3 cg12533010 | 145 | 1.73E-03 | 1.50E-02 | 3.43 (0.67) | 4.34 (0.55) | 1/11 | 0.6 (0.03) ^a^ |
| PCGF6 cg26155979 | 25 | 1.77E-03 | 1.00E-03 | 32.37 (3.77) | 28.97 (0.6) | 1/4 | -0.06 (0.44) |
| GPR155 cg14596623 | -86 | 1.81E-03 | 3.31E-03 | 14.65 (2.4) | 11.35 (1.47) | 3/14 | 0.03 (0.46) |
| SEPT5 cg19940065 | -252 | 1.84E-03 | 1.99E-02 | 11.09 (1.97) | 9.16 (1.25) | 1/9 | 0.45 (0.09) |
| BEND5 cg06744574 | 188 | 1.88E-03 | 1.27E-03 | 6.66 (1.24) | 5.65 (0.17) | 1/8 | 0.13 (0.36) |
| RING1 cg08892712 | -185 | 1.88E-03 | 5.04E-03 | 6.92 (1.51) | 5.62 (0.76) | 2/36 | N/A |
| LOC100129361 cg17844553 | -1168 | 1.89E-03 | 1.07E-03 | 93 (1.63) | 94.2 (0.48) | 1/2 | N/A |
| FCHO2 cg18049045 | -1291 | 1.89E-03 | 6.18E-04 | 92.22 (1.74) | 93.7 (0.57) | 1/10 | 0.34 (0.16) |
| LOC100507557 cg12831148 | 82 | 1.89E-03 | 1.19E-02 | 4.5 (0.46) | 5.3 (0.6) | 1/9 | N/A |
| HCFC2 cg21917950 | 871 | 1.90E-03 | 1.11E-02 | 21.54 (1.79) | 23.2 (1.39) | 1/3 | -0.43 (0.11) |
| ACBD6 cg08270031 | -185 | 1.91E-03 | 1.13E-03 | 8.75 (1.18) | 9.48 (0.32) | 3/11 | -0.58 (0.04) ^a^ |
| IRX6 cg01554410 | -13 | 1.91E-03 | 7.98E-04 | 21.42 (2.93) | 19.84 (0.57) | 2/9 | 0.08 (0.41) |
| AX746851 cg18833573 | -1871 | 1.94E-03 | 2.59E-03 | 11.83 (1.93) | 10.02 (1.25) | 2/10 | N/A |
| ZNFX1 cg20907517 | 0 | 1.95E-03 | 1.47E-05 | 12.5 (2.12) | 10.56 (0.53) | 2/5 | 0.04 (0.45) |
| HSPA2 cg01141043 | -135 | 1.95E-03 | 1.26E-04 | 11.78 (3.11) | 9.3 (0.56) | 2/22 | 0.55 (0.05) ^a^ |
| KLLN cg22564317 | 724 | 1.96E-03 | 6.96E-03 | 5.59 (0.45) | 6.48 (0.44) | 5/50 | N/A |
| KRT8 cg10843343 | 841 | 1.97E-03 | 7.03E-03 | 59.58 (5.4) | 55.88 (1.46) | 2/21 | -0.26 (0.23) |
| TP53BP1 cg17859659 | -15 | 1.97E-03 | 1.04E-04 | 5.63 (1.62) | 4.17 (0.69) | 2/20 | 0.07 (0.43) |
| WSCD2 cg15873673 | 87 | 1.97E-03 | 5.17E-03 | 12.11 (0.79) | 13.25 (0.65) | 6/15 | N/A |
| MSH5-SAPCD1 cg23162518 | -352 | 1.99E-03 | 1.66E-02 | 68.21 (3.5) | 64.67 (2.57) | 1/7 | N/A |
| UBE2Q2 cg04672495 | 491 | 2.00E-03 | 5.42E-04 | 12.81 (1.59) | 14.63 (1.47) | 1/7 | 0.56 (0.04) ^a^ |
| NKX2-1 cg01566235 | -1671 | 2.02E-03 | 5.30E-03 | 11.4 (1.26) | 12.62 (0.65) | 3/9 | -0.44 (0.10) |
| RARG cg13937905 | 1646 | 2.04E-03 | 5.89E-03 | 87.6 (5.47) | 84.58 (1.07) | 1/18 | -0.44 (0.10) |
| TMEM138 cg18595258 | -900 | 2.05E-03 | 5.82E-04 | 75.59 (2.59) | 76.67 (1.46) | 1/10 | -0.22 (0.27) |
| LOC100498859 cg12151942 | -1882 | 2.05E-03 | 9.68E-03 | 73.25 (3.51) | 76.4 (2.13) | 1/9 | N/A |
| NCALD cg27637873 | -1239 | 2.06E-03 | 5.18E-05 | 90.87 (2.5) | 89.65 (0.18) | 2/14 | -0.08 (0.41) |
| LAT cg23797100 | -93 | 2.07E-03 | 1.08E-04 | 3.64 (0.61) | 4.49 (0.17) | 1/13 | 0.18 (0.30) |
| PNMT cg01266707 | -45 | 2.08E-03 | 6.04E-04 | 33.66 (4.37) | 30.41 (2.36) | 1/5 | -0.19 (0.30) |
| MAP2K3 cg25677314 | 1065 | 2.10E-03 | 1.24E-03 | 84.12 (2.38) | 82.39 (0.51) | 2/18 | 0.41 (0.12) |
| KDELR2 cg08622675 | -1149 | 2.12E-03 | 4.71E-03 | 98.08 (0.6) | 98.59 (0.22) | 1/11 | 0.27 (0.22) |
| PTMS cg08102602 | 102 | 2.12E-03 | 7.17E-03 | 6.34 (1.14) | 5.62 (0.37) | 1/5 | -0.35 (0.16) |
| AP4S1 cg15301694 | 1006 | 2.16E-03 | 1.14E-02 | 92.44 (1.42) | 91.62 (1.31) | 1/5 | -0.06 (0.44) |
| SLC4A8 cg19265103 | -487 | 2.17E-03 | 1.94E-05 | 23.04 (2.63) | 25.44 (0.54) | 2/19 | -0.52 (0.06) |
| TDH cg25447652 | 601 | 2.17E-03 | 2.21E-03 | 5.29 (1.49) | 5.99 (0.52) | 3/10 | -0.39 (0.13) |
| DPP9 cg18347423 | 241 | 2.17E-03 | 1.19E-03 | 6.34 (1.57) | 4.74 (0.71) | 1/5 | -0.44 (0.10) |
| PIP4K2B cg27142680 | -631 | 2.17E-03 | 2.85E-03 | 63.03 (3.22) | 65.66 (1.61) | 1/6 | -0.12 (0.38) |
| SNRPF cg01090661 | -124 | 2.19E-03 | 1.30E-02 | 2.65 (0.79) | 3.12 (0.48) | 2/17 | 0.04 (0.45) |
| HIVEP3 cg02031397 | 898 | 2.20E-03 | 2.28E-04 | 8.34 (1.83) | 7.05 (0.1) | 1/19 | -0.26 (0.23) |
| TMEM41A cg01892655 | 299 | 2.20E-03 | 1.31E-03 | 11.1 (2.41) | 9.11 (1.27) | 1/12 | -0.34 (0.17) |
| FOXP4 cg11436430 | -59 | 2.21E-03 | 1.52E-02 | 3.35 (0.96) | 2.8 (0.33) | 2/11 | -0.31 (0.19) |
| MYO5A cg10535933 | -171 | 2.21E-03 | 1.82E-04 | 7.77 (1.46) | 6.47 (0.5) | 2/13 | 0.31 (0.19) |
| DLGAP4 cg26330133 | -3 | 2.22E-03 | 1.47E-03 | 8.05 (1.23) | 8.71 (0.49) | 2/18 | 0.25 (0.25) |
| COX18 cg04396098 | -177 | 2.26E-03 | 1.52E-02 | 5.66 (0.52) | 6.45 (0.7) | 1/12 | -0.25 (0.24) |
| DNMT3A cg00912598 | -107 | 2.26E-03 | 4.55E-03 | 9.56 (1.9) | 11.31 (0.93) | 1/31 | 0.33 (0.18) |
| REEP5 cg09835486 | 63 | 2.26E-03 | 9.92E-03 | 7.74 (1.34) | 6.41 (0.7) | 1/8 | 0.37 (0.15) |
| ATP6V1C2 cg00454409 | -408 | 2.26E-03 | 1.85E-02 | 7.82 (1.66) | 6.8 (0.7) | 3/7 | 0.11 (0.38) |
| SLC12A5 cg12832857 | 876 | 2.27E-03 | 1.18E-02 | 11.22 (2.84) | 9.6 (1.35) | 1/15 | 0.5 (0.07) |
| C14orf37 cg19764854 | 654 | 2.28E-03 | 3.66E-03 | 9.17 (0.78) | 9.96 (0.46) | 2/12 | -0.44 (0.1) |
| SLC27A3 cg15930240 | 1248 | 2.35E-03 | 4.82E-03 | 90.55 (1.1) | 89.17 (0.93) | 1/13 | -0.42 (0.11) |
| RBM11 cg13817547 | 383 | 2.35E-03 | 1.31E-04 | 5.38 (0.91) | 6 (0.3) | 1/8 | 0.1 (0.39) |
| CCND1 cg25060573 | 1249 | 2.36E-03 | 1.91E-03 | 7.12 (0.87) | 6.52 (0.6) | 2/31 | -0.4 (0.13) |
| SGSM2 cg19216660 | -1219 | 2.36E-03 | 4.05E-04 | 98.33 (0.26) | 98.55 (0.13) | 1/9 | -0.57 (0.04) |
| ZMYND19 cg00466158 | 90 | 2.37E-03 | 5.58E-04 | 2.34 (0.21) | 1.95 (0.13) | 2/4 | -0.29 (0.21) |
| CDC42EP2 cg07477137 | 1044 | 2.40E-03 | 4.47E-04 | 8.54 (1.19) | 9.92 (0.71) | 2/14 | 0.26 (0.23) |
| BMP4 cg02254554 | -123 | 2.41E-03 | 1.80E-02 | 10.18 (1.75) | 9.18 (0.64) | 2/20 | -0.04 (0.46) |
| WDR70 cg26903222 | -1182 | 2.41E-03 | 1.08E-03 | 83.1 (2.07) | 84.62 (1.54) | 1/7 | 0.29 (0.20) |
| KCNK3 cg01805869 | 192 | 2.42E-03 | 6.63E-03 | 24.26 (3.22) | 21.28 (0.94) | 1/9 | 0.09 (0.40) |
| SGK196 cg23110471 | -104 | 2.42E-03 | 1.30E-03 | 5.23 (0.88) | 4.29 (0.32) | 2/10 | 0.28 (0.21) |
| FAM133B cg27519869 | -99 | 2.43E-03 | 1.99E-03 | 5.1 (0.45) | 5.88 (0.31) | 2/11 | 0.37 (0.15) |
| AX746771 cg07428439 | 141 | 2.45E-03 | 1.32E-02 | 15.97 (2.36) | 13.65 (1.57) | 1/2 | N/A |
| BC040577 cg03399598 | -199 | 2.48E-03 | 6.80E-04 | 9.58 (0.9) | 10.87 (0.46) | 1/4 | N/A |
| ZBTB24 cg20560186 | 337 | 2.48E-03 | 1.13E-03 | 8.87 (2.08) | 7.99 (0.81) | 2/4 | -0.15 (0.34) |
| MB21D1 cg08905652 | 944 | 2.49E-03 | 3.96E-04 | 4.42 (0.77) | 4.9 (0.32) | 1/11 | N/A |
| OLIG3 cg03889226 | 198 | 2.50E-03 | 1.34E-03 | 8.01 (1.35) | 6.72 (0.49) | 2/13 | -0.23 (0.26) |
| LPAR2 cg16692998 | -134 | 2.52E-03 | 2.39E-03 | 11.66 (2.21) | 9.78 (1.04) | 3/16 | 0.25 (0.24) |
| TNFAIP3 cg22466620 | -728 | 2.52E-03 | 2.73E-03 | 13.18 (2.47) | 10.98 (1.24) | 3/14 | -0.14 (0.35) |
| RB1CC1 cg22122414 | -41 | 2.52E-03 | 6.95E-04 | 7.84 (1.23) | 6.19 (0.56) | 1/10 | -0.46 (0.09) |
| AGTR1 cg04878152 | 303 | 2.55E-03 | 1.69E-02 | 19.02 (3.03) | 17.48 (1.26) | 2/10 | -0.39 (0.13) |
| UGP2 cg00626599 | -108 | 2.56E-03 | 1.69E-02 | 7 (0.61) | 8.27 (0.61) | 3/24 | 0.11 (0.38) |
| PRMT10 cg02715115 | -344 | 2.57E-03 | 7.09E-03 | 4.87 (0.64) | 5.66 (0.27) | 1/11 | 0.13 (0.36) |
| MXD4 cg05124756 | -81 | 2.57E-03 | 9.10E-04 | 7.49 (1.23) | 6.42 (0.62) | 4/13 | 0.33 (0.18) |
| RAB20 cg26410311 | -116 | 2.58E-03 | 1.45E-02 | 9.08 (2.39) | 7.61 (0.93) | 1/7 | 0.39 (0.13) |
| MVP cg08168844 | -741 | 2.62E-03 | 1.70E-05 | 92.12 (0.9) | 90.71 (0.43) | 1/4 | -0.57 (0.04) ^a^ |
| ERCC3 cg13422881 | -58 | 2.63E-03 | 1.25E-02 | 6.85 (1.58) | 5.57 (0.97) | 3/10 | -0.11 (0.38) |
| NR2E1 cg07411432 | -114 | 2.63E-03 | 6.79E-03 | 15.66 (1.07) | 14.13 (0.95) | 3/19 | 0.25 (0.24) |
| OSM cg14519350 | 880 | 2.63E-03 | 2.06E-03 | 7.9 (1.1) | 8.92 (0.26) | 2/8 | 0.16 (0.32) |
| TBC1D12 cg11821440 | -7 | 2.63E-03 | 2.83E-03 | 4.43 (0.98) | 3.43 (0.42) | 1/14 | -0.16 (0.32) |
| LOC286083 cg03964343 | -279 | 2.64E-03 | 1.77E-02 | 87.71 (1.31) | 86.82 (0.86) | 1/4 | N/A |
| MAP6 cg04371288 | 979 | 2.70E-03 | 1.18E-03 | 1.42 (0.47) | 1.53 (0.17) | 3/15 | 0.28 (0.21) |
| CELF4 cg11154070 | 17 | 2.70E-03 | 5.60E-04 | 9.87 (1.51) | 11.04 (0.54) | 2/37 | -0.5 (0.07) |
| COQ4 cg11980791 | 7 | 2.70E-03 | 7.81E-03 | 4.66 (0.46) | 5.39 (0.47) | 1/4 | 0.08 (0.41) |
| FGFR1OP2 cg17643699 | 180 | 2.72E-03 | 2.32E-03 | 10.15 (2.45) | 8.23 (1.32) | 1/8 | 0.38 (0.14) |
| LYZL4 cg21044104 | 189 | 2.73E-03 | 3.63E-03 | 79.51 (0.98) | 81.14 (1.06) | 1/4 | -0.4 (0.13) |
| SEC14L1 cg25344639 | 140 | 2.74E-03 | 9.52E-06 | 10.06 (1.25) | 9.37 (0.43) | 3/25 | -0.51 (0.06) |
| ADPRHL1 cg05314639 | -1890 | 2.75E-03 | 1.70E-03 | 90.55 (0.94) | 90.96 (0.46) | 1/23 | -0.02 (0.48) |
| BC030591 cg10747529 | -597 | 2.77E-03 | 3.60E-03 | 24.54 (3.79) | 23.33 (1.75) | 1/5 | N/A |
| PPT2-EGFL8 cg04264374 | 62 | 2.77E-03 | 1.16E-03 | 4.15 (0.48) | 4.61 (0.37) | 1/4 | N/A |
| MED21 cg02566159 | -14 | 2.79E-03 | 3.23E-03 | 3.3 (0.49) | 3.57 (0.33) | 2/8 | -0.24 (0.25) |
| HABP4 cg14041153 | 65 | 2.80E-03 | 7.54E-03 | 8.74 (1.72) | 7.5 (0.79) | 1/5 | -0.56 (0.04) ^a^ |
| MAML1 cg03899909 | -190 | 2.82E-03 | 7.36E-04 | 14.58 (2.73) | 12.58 (0.62) | 2/10 | -0.18 (0.31) |
| IDH1 cg26105702 | -389 | 2.84E-03 | 3.12E-04 | 12.33 (1.73) | 10.39 (0.85) | 1/11 | -0.43 (0.11) |
| TMUB1 cg03519451 | 96 | 2.84E-03 | 4.57E-04 | 11.55 (1.35) | 10.63 (0.65) | 1/18 | 0.24 (0.25) |
| CCDC57 cg08576197 | 67 | 2.85E-03 | 1.72E-03 | 7.45 (0.85) | 8.73 (0.4) | 1/5 | -0.33 (0.17) |
| HEPACAM cg06142324 | 770 | 2.85E-03 | 9.19E-03 | 92.19 (1.25) | 91.27 (0.56) | 1/4 | 0.08 (0.42) |
| RGS7 cg03613077 | -426 | 2.86E-03 | 7.73E-03 | 6.68 (1.56) | 5.48 (0.67) | 3/12 | 0.42 (0.11) |
| C19orf25 cg18717146 | 513 | 2.87E-03 | 1.68E-02 | 22.88 (3.42) | 25.05 (1.28) | 2/10 | -0.23 (0.26) |
| TATDN3 cg06593568 | 412 | 2.87E-03 | 1.20E-03 | 2.66 (0.52) | 3.45 (0.41) | 1/4 | N/A |
| FRYL cg27297724 | 405 | 2.88E-03 | 1.62E-03 | 2.05 (0.45) | 1.69 (0.07) | 2/12 | 0.3 (0.20) |
| PAPOLG cg05731991 | -67 | 2.90E-03 | 1.54E-02 | 7.79 (1.11) | 6.6 (1.01) | 1/7 | 0.27 (0.22) |
| PHF21B cg20525183 | 41 | 2.93E-03 | 1.33E-02 | 11.49 (2.35) | 9.75 (0.92) | 4/24 | -0.58 (0.04) ^a^ |
| LOC100129726 cg15039414 | 338 | 2.95E-03 | 7.14E-03 | 7.21 (1.29) | 6.15 (0.52) | 2/19 | 0.33 (0.17) |
| PIGY cg17714987 | -520 | 2.95E-03 | 1.58E-02 | 57.76 (5.47) | 59.73 (2.04) | 1/10 | 0.48 (0.08) |
| MIR4750 cg23074703 | 1490 | 2.98E-03 | 1.27E-03 | 92.78 (2.85) | 91.52 (0.84) | 2/5 | N/A |
| PIGL cg05310071 | 61 | 2.98E-03 | 2.95E-03 | 3.24 (0.95) | 4.18 (0.43) | 1/8 | 0.23 (0.26) |
| PRR24 cg11557938 | -35 | 2.99E-03 | 1.49E-03 | 5.2 (2.39) | 3.15 (0.43) | 3/14 | N/A |
| FAM107A cg04047146 | 1897 | 3.02E-03 | 2.04E-02 | 21.79 (3.7) | 23.33 (1.72) | 1/10 | 0.07 (0.42) |
| SLC25A42 cg18420266 | -35 | 3.03E-03 | 1.83E-02 | 10.41 (1.57) | 8.92 (1) | 1/11 | -0.21 (0.28) |
| FDX1L cg00791249 | -31 | 3.03E-03 | 3.70E-03 | 6.85 (0.81) | 6.48 (0.23) | 1/5 | -0.17 (0.32) |
| ADCY9 cg26952925 | -205 | 3.04E-03 | 1.02E-02 | 2.28 (0.64) | 1.64 (0.25) | 1/10 | -0.09 (0.40) |
| FBRSL1 cg11228480 | -645 | 3.06E-03 | 9.19E-03 | 20.34 (4.18) | 16.62 (2.46) | 6/33 | 0.37 (0.14) |
| SMTN cg07006526 | -228 | 3.06E-03 | 8.67E-03 | 1.69 (0.36) | 1.27 (0.29) | 2/16 | -0.28 (0.21) |
| NOTCH4 cg11747349 | 818 | 3.09E-03 | 5.75E-03 | 13.23 (2.91) | 11.74 (1.67) | 5/62 | -0.08 (0.41) |
| RABIF cg24744425 | 232 | 3.10E-03 | 1.14E-03 | 1.87 (0.2) | 1.55 (0.13) | 1/12 | 0.28 (0.22) |
| ZDHHC24 cg06796806 | 133 | 3.10E-03 | 1.24E-03 | 1.32 (0.42) | 1.09 (0.1) | 1/8 | 0.2 (0.29) |
| TRIM47 cg03700492 | 33 | 3.12E-03 | 2.95E-03 | 7.06 (0.58) | 8.06 (0.18) | 1/11 | -0.76 (<0.01) ^a^ |
| HSPA4 cg24159723 | -22 | 3.13E-03 | 7.53E-03 | 16.31 (2.24) | 14.73 (1.1) | 1/12 | 0.24 (0.26) |
| CCDC140 cg15825116 | 965 | 3.15E-03 | 8.62E-04 | 6.32 (1.07) | 7.47 (0.57) | 2/23 | 0.2 (0.29) |
| USP2 cg03263549 | -58 | 3.16E-03 | 9.37E-04 | 16.28 (2.91) | 13.59 (1.17) | 1/11 | 0.22 (0.27) |

**Legend:** Probes are defined by their closest gene and location in the genome. Location is given in base pairs relative to the transcription start site. A negative value in this column denotes number of base pairs upstream of the TSS. Step I P-value refers to the probability that coefficient "a" in equation 1 is zero. The cutoff in this step was p<10^-2.5^. Step II p-values are stem from a t-test between the TSD induced change and the median change in technical replicates (Benjamini-Hochberg correction). Averaged methylation beta values in % and their standard deviations are given for both conditions. The second to last column indicates the number of nominally significant probes (p<0.05) associated with a given gene along with the total number of probes annotated to the same gene. Finally, a correlation coefficient between methylation of the probe and expression of the closest gene in the separate cohort of whole blood (E-GEOD-49065) is listed together with the p-value for the correlation test. Significant methylation-expression correlations are marked with "*".
